# Preparation of Cell-Loaded Microbeads as Stable and Injectable Delivery Platforms for Tissue Engineering

**DOI:** 10.3390/biomimetics8020155

**Published:** 2023-04-13

**Authors:** Mehmet Ali Karaca, Derya Dilek Kancagi, Ugur Ozbek, Ercument Ovali, Ozgul Gok

**Affiliations:** 1 Department of Medical Biotechnology, Institute of Health Sciences, Acibadem Mehmet Ali Aydinlar University, Istanbul 34752, Turkey; 2 Acibadem Labcell Cellular Therapy Laboratory, Istanbul 34752, Turkey; 3 Medical Genetics Department, School of Medicine, Acibadem Mehmet Ali Aydinlar University, Istanbul 34752, Turkey; 4 Department of Biomedical Engineering, Faculty of Engineering and Natural Sciences, Acibadem Mehmet Ali Aydinlar University, Istanbul 34752, Turkey

**Keywords:** cell delivery, cell-loaded beads, microbeads, encapsulation

## Abstract

Cell transplants in therapeutic studies do not preserve their long-term function inside the donor body. In mesenchymal stem cell (MSC) transplants, transplanted cells disperse through the body and are prone to degradation by immune cells after the transplant process. Various strategies, such as usage of the immunosuppressive drugs to eliminate allograft rejection, are designed to increase the efficiency of cell therapy. Another strategy is the construction of biomimetic encapsulates using polymeric materials, which isolate stem cells and protect them from environmental effects. In this study, fibroblasts (L929) and MSCs were investigated for their improved viability and functionality once encapsulated inside the alginate microbeads under in vitro conditions for up to 12 days of incubation. Thus, uniform and injectable (<200 µm) cell-loaded microbeads were constructed by the electrostatically assisted spraying technique. Results showed that both L929 and MSCs cells continue their metabolic activity inside the microbeads during the incubation periods. Glucose consumption and lactic acid production levels of both cell lines were consistently observed. The released cell number on day 12 was found to be increased compared to day 0. Protein expression levels of both groups increased every day with the expected doubling rate. Hence, this strategy with a simple yet clever design to encapsulate either MSCs or L929 cells might outstand as a potential cell delivery platform for cell therapy-based tissue engineering.

## 1. Introduction

Mesenchymal stem cells (MSCs) are a component of the tissue construct, which secrete growth hormones or factors and regulate the signaling pathway via secretion of the various cytokines and growth factors, such as vascular endothelial growth factors and transforming growth factor (TGF-α). The injection of MSCs into the degenerative tissue lead to the secretion of therapeutic proteins, construction, and regeneration of the tissue [1,2]. Mesenchymal stem cells have been widely used to provide effective treatment for diseases such as cardiovascular disease, orthopedic disease, rheumatologic disease, endocrine disease, and neurodegenerative disease [3]. The encapsulation of MSCs via various polymeric materials enhances their therapeutic efficiency for transplantation without applying immunosuppressive drugs [4,5]. A recent study has illustrated that rat adipose-derived stem cells express osteogenic factors in vitro and regenerate bone fractures in vivo [5,6].

Mesenchymal stem cells (MSCs) disperse through the tissue after the transplantation of the cells [7]. Injection of the MSCs induces an immunological response that decreases the cellular activity of transplanted cells in damaged tissue [8]. Suspended formation of the MSCs limits the differentiation of the cells and expression of the regenerative factors [9]. Therefore, MSCs require a vascularized 3D construct for more efficient cellular therapy. MSC-loaded alginate microbeads provide 3D constructs and a large surface area for cell proliferation and attachment. So, encapsulation of MSCs might eliminate the dispersion of the cells inside the tissue after the injection procedure.

The most commonly used biomaterial for the encapsulation process is the natural polymer Alginate is a negatively charged polysaccharide and is used in various applications, such as biotechnological industries, pharmaceuticals, food, and textiles [10,11]. A sequence of the alginate containing α-l-guluronic acid (G) and β-d-mannuronic acid side (M) characterizes the functionality and gelation of alginate. On the other hand, alteration of the environmental factors, such as the ionic strength of the medium, the concentration of gelling ions, and the pH of the solution, regulate the gelation behavior of alginate polymer chains. For example, the deprotonation of carboxylic acid groups in alginate structure at a certain pH value ameliorates its dissolving rate. Sodium alginate might become insoluble and then precipitate as alginic acid in the solution by increasing the pH value. A three-dimensional network of the alginate is obtained by ionic bonding via the diffusion of multivalent cations through the polymer chains. Similarly, ionic bonding between the multivalent cations and alginate might break down with releasing of these cations by either dilution or pH change of the microenvironment, so the alginate-based gel form gets disrupted [12].

Various encapsulation strategies, such as drop generation by gravity electrostatically assisted spraying, and aerodynamically assisted jetting systems are used to prepare microbeads form. Leslie et al. have shown that the injectable size of adipose mesenchymal stem cell-loaded alginate microbeads might be constructed with an electrostatically assisted spraying system. Adipose mesenchymal stem cell-loaded alginate microbeads by electrostatically assisted spraying system were seen to regenerate bone fraction post-transplant week 2 [13]. Additionally, allogenic islet transplantation was used as an effective procedure for the regulation of insulin hormone levels inside the body. Islet-loaded alginate microbeads continue their functionality in patients with type 1 diabetes at post-transplant week 1 [14]. Moreover, co-encapsulation of the islet cells with MSCs increases the nitric oxide production rate and secretion of immunomodulatory cytokines inside the tissue, so the immunomodulatory activity of the MSC was shown to enhance the efficiency of islet transplantation [15].

Various physiological (pH, enzymatic activity, redox potential, and glucose concentration) and external stimuli (mechanical forces, light, temperature, and magnetic field) control the degradation rate of polysaccharides. Obviously, alterations in pH value significantly affect the ionic strength of polymeric materials. Alginate chains stabilize their structure at pH values ranging from 5 to 10. Its degradation by environmental pH values has occurred via β-alkoxy-elimination (pH > 10) and proton catalyzed by hydrolysis (pH < 5) [16]. Polymers might be naturally degraded by the upregulation of enzymes on the polymeric backbone, whereas alginate is naturally degraded by alginate lysates and alginate polymerases, which are not synthesized inside the human body. This mechanism was introduced by the β-elimination mechanism [17]. The other degradation mechanism for alginate microbeads is the non-enzymatic breakdown of bonds between the polymer chains via Ca^2+^-based chelating by chemical reagents such as EDTA and sodium citrate. Calcium content inside the alginate microbeads is not preserved in in vitro conditions because of the cell proliferation inside the microbeads and the diffusion of cellular metabolites (lactate, phosphate, and citrate) to the environment. Releasing the calcium from the microbeads also affects the mechanical property of the alginate microbeads by causing the disintegration of polymer chains and subsequent deformation.

Herein, cell-loaded microbeads were prepared by using an encapsulator to obtain an efficient delivery platform for living cells in cellular therapy-based applications. In addition, the effect of cell density (L929 and human MSCs) in the alginate microbeads was evaluated on cell proliferation, the morphology of alginate microbeads, and the metabolite production rate for encapsulated cells. For that purpose, cell-loaded alginate microbeads were fabricated with an electrostatically assisted spraying system and then monitored under the fluorescence microscope during 12 days of incubation. The number of released cells from microbeads, their viability, and the production rate of metabolites (glucose consumption, lactic acid production, and protein production) were analyzed and reported as an enlightening optimization procedure for the preparation of promising biomaterial-based cell-delivery platforms.

## 2. Materials and Methods

### 2.1. Materials

Alginate (50% mannuronate units, Sigma-Aldrich, low viscosity, A1112, St. Louis, MO, USA) solution was prepared as 4% by weight (*w*/*v*) in saline (0.9% isotonic sodium chloride/Polypharma/Polyfleks) solution and stirred overnight at room temperature. Calcium chloride (CaCl_2_) (Merck, Darmstadt, Germany) (75 mM) was used as a cross-linker. Nozzles (inner diameter: 0.35 mm) were incubated inside sodium citrate solution (85 mM) to prevent clogging of the system. Encapsulation systems were sterilized with ethanol (70% *v*/*v*) spraying. The system was exposed to UV light for 2 h before being used for cell culture experiments.

### 2.2. Preparation and Optimization of Microbeads

Ethanol (70% *v*/*v*) was run before the operation of the system (Nisco Encapsulator VAR V1 LIN-0043, Nisco Engineering AG, Zurich, Switzerland). UV-sterilized research-grade alginate solution was loaded into a 50 mL syringe, and the syringe pump was set at a flow rate of 5 mL/h. Stirrer speed was adjusted to the desired level. Operation arm and nozzle tip (0.35 mm) were installed inside the system. Then, the system was run with alginate solution until loading of the system (cables and tip). The electrode was dipped inside the cross-linking solution, and voltage was adjusted after dripping of alginate solution into the cross-linking solution. Alginate microbeads were fabricated by applying desired electrostatic force (6 kVa), which is an optimum value for microbead production [18]. The voltage and syringe pump were closed after 30 min of alginate flow. Various concentrations of microbead groups (5000, 2500, 1250, 500, and 250 bead/mL) were incubated in 1 mL MSC nutrient medium. The medium was exchanged before the measurement of the microbead diameter at each time point.

Morphological evolutions of microbeads were visualized using a fluorescence microscope (Zeiss Axio Vert.A1 inverted microscope, Oberkochen, Germany) for advanced routine. A total of 3 microbeads were selected for each group, and the diameter of the microbeads was measured at days 0, 1, 3, 7, and 14 using Zeiss Program (Carl Zeiss Microscope, Oberkochen, Germany). The diameter values of microbeads were graphed using Microsoft Excel Program.

Stability of microbeads in various conditions, their sizes, and morphological structure were evaluated at various environmental conditions (Acetate solution, Medium solution (Nutrient Free Medium (Biological Industries, Beit HaEmek, Israel)), Phosphate Buffer Solution (PBS, Gibco, New York, NY, USA). Acetate solution (100 mM) was prepared with powder sodium acetate and desired pH was adjusted to 5.5 using 1 M HCl and 1M NaOH stock solution.

After 1 and 5 days of the experiment, microbeads were visualized using a fluorescence microscope (Zeiss Axio Vert.A1 inverted microscope). A total of 3 microbeads were selected for each group, and the diameter of the microbeads was measured using Zeiss Program at days 0, 1, 3, 7, and 14 (Carl Zeiss Microscope, Oberkochen, Germany). The diameters of microbeads were graphed using Microsoft Excel Program.

### 2.3. Preparation of Cell-Loaded Microbeads

Fibroblast cells (L929, ATCC, NCTC clone 929) were cultured in MSC nutrient-free medium (Biological Industries, Beit HaEmek, Israel). After thawing of cells according to the manufacturing procedure, cells (5 × 10^4^ cells/cm^2^) were seeded in a T-25 flask and incubated at 37 °C and 5% CO_2_. The waste medium was removed, and a fresh medium was supplied every 48 h. When cells reached 80% confluence, they were trypsinized and centrifuged at 300 RCF for 5 min. Cells were collected in ringer lactate solution (0.5% Human Serum Albumin Solution (Octapharma, Switzerland)) and calculated with a cell counter device (BioRad).

Adipose stem cells were cultured in MSC nutrient-free medium with 100 IU/mL penicillin and 100 µg/mL streptomycin (Gibco, New York, NY, USA). After thawing of the MSCs, they (2 × 10^4^ cells/cm^2^) were seeded in a T-25 flask. Tissue culture dishes were incubated at 37 °C and 5% CO_2_.The waste medium was removed, and a fresh medium was supplied every 48 h. When cells reached 80% confluency, they were trypsinized and centrifuged 300 RCF for 10 min. Cells were collected in ringer lactate solution (0.5% Human Serum Albumin Solution) and calculated with a cell counter device (BioRad).

L929 cells and MSCs were trypsinized from a Petri dish and counted with a cell counter (BioRad cell counter). L929 cells (20 × 10^6^ cells) and MSCs (10 × 10^6^ cells) were used for loading into alginate microbeads. For sterilization of the system, ethanol (70% *v*/*v*) was run before the operation of the system (Nisco Encapsulator VAR V1 LIN-0043, Nisco Engineering AG, Zurich, Switzerland).The system was treated with UV light (2 h) before the cell culture experiments. Cells were diluted into 4 × 10^6^ cells/mL (L929) (~48 cells in 1 microbead) and 2 × 10^6^ cells (MSCs) (~24 cells in 1 microbead) with UV sterilized alginate polymer solution and put into the syringe (Becton Dickinson (BD), East Rutherford, NJ, USA).

Then, the system was run with a cell–alginate mixture until full loading of the system (cables and 0.35 mm tip) with it. The electrode was dipped into the cross-linking solution, and voltage was adjusted afterwards. Cell-loaded alginate microbeads were fabricated by applying desired electrostatic force (6 kVa), which is an optimum value for microbead production [19]. The voltage and syringe pump were closed after 30 min of the running of alginate solution.

Microbeads were washed 2 times with saline solution (0.9% isotonic sodium chloride/Polypharma/Polyfleks (Istanbul, Turkey)).Various concentrations of the microbeads were cultured in a mesenchymal stem cell medium (MSCs nutrient-free medium, Biological Industries). Each group (Mesenchymal stem cell (400, 200, 100, and 50 bead/mL), L929 (1000, 500, 250, 125 bead/mL) were transferred to (2 × T25) flask for the cultivation of the cell loaded microbeads.

### 2.4. Characterization of Cell-Loaded Microbeads

*Functional Group Analysis*: Chemical structures of the obtained alginate microbeads were confirmed by Fourier-transform infrared spectroscopy (FT-IR, Thermo Fisher Scientific Inc. (Waltham, MA, USA); Nicolet 380), and result was compared with that of pure alginate polymer and literature data.

*Mechanical evaluation*: Obtained alginate microbeads were analyzed for their mechanical properties by Malvern Kinexus rheometer using J2 SR 4703 SS geometry. Degradation behavior of microbeads was studied between γ = 0.001 and γ = 1 at f = 1 Hz at 25 °C.

*Morphological evaluation*: Culture medium was discarded, and fresh medium was added into microbeads environment at each time point of the experiment. Microbeads were visualized with bright field microscope after addition of fresh medium (MSC NutriStem^®^ XF Medium), and the diameter of the microbeads was measured using Zeiss Program (Carl Zeiss Microscope). The diameter value of each group was graphed using Microsoft Excel. Cells were also visualized via a fluorescence microscope (Zeiss Axio Vert.A1 inverted microscope for advanced routine). GFP signal was measured using the ImageJ program, and the intensity values of the microbeads were graphed with Microsoft Excel.

### 2.5. Glucose and Lactic Acid Measurements

The metabolic activity of the cells inside the microbeads was tracked during 12-day incubation. Glucose consumption and lactic acid production inside the cultivation environment were measured, where obtained results provided information about the cellular metabolic activity. Cell medium (500 µL sample for glucose and 500 µL sample for lactic acid) was collected for 12 days. The collected cultivation medium was analyzed with the ADVIA^®^ 1800 Clinical Chemistry System (Berlin, Germany). In this system, reagents were loaded into the system before their usage. The concentration of the glucose and lactic acid levels inside the solution was measured with ADVIA^®^ 1800 Clinical Chemistry System (Berlin, Germany) [19]. Glucose consumption and lactic acid production of the cells encapsulated into the microbeads were graphed using Microsoft Excel.

### 2.6. Cell Viability in Culture Medium

Cell culture medium was collected after 12 days of incubation. Attached cells to the flask surface were removed by being trypsinized and centrifuged at 300 RCF for 10 min. Cells were collected in ringer lactate solution (0.5% Human Serum Albumin Solution) and calculated with a cell counter device (BioRad (Hercules, CA, USA)). The viability of cells was assessed with Trypan blue staining protocol.

### 2.7. Total Protein and mRNA Concentration in Culture Medium

Cell medium (1 mL sample cultivation medium) was collected during the incubation period for microbeads. The total protein amount in the solution was measured with a BCA assay kit (Takara, Shiga, Japan). BCA assay was performed according to the manufacturing procedure. After incubation of the reagent with the sample, the concentration of the total protein in the medium was calculated by measuring the absorbance taken at 562 nm via a microplate reader. Measurement of the absorbance value was graphed using Microsoft Excel.

### 2.8. Nanodrop Measurement

Cell medium (1 mL sample cultivation medium) was collected during the incubation period for microbeads. The amount of total mRNA in the sample tube was measured by Thermo Scientific Nanodrop One (Waltham, MA, USA). Measurement of the absorbance value was graphed using Microsoft Excel.

### 2.9. SDS PAGE

Protein samples in a cell culture medium were collected at the end of 12-day incubation. Samples were loaded on SDS Page (10%). SDS Page was stained with Coomassie brilliant blue dye. Images were observed and analyzed with BIO-RAD ChemiDoc XRS+ Molecular Imager with Lab Software (Hercules, CA, USA).

### 2.10. Statistical Analysis

Statistical analyses were performed using Microsoft Excel software (Redmond, WA, USA). Obtained results were analyzed by running a Student’s *t*-test from the averaged data obtained from 3 independent experiments with a *p*-value < 0.05. Levels of significance were shown at *: *p* < 0.05, **: *p* < 0.01, and ***: *p* < 0.001.

## 3. Results

### 3.1. Microbead Fabrication

**Figure 1:**
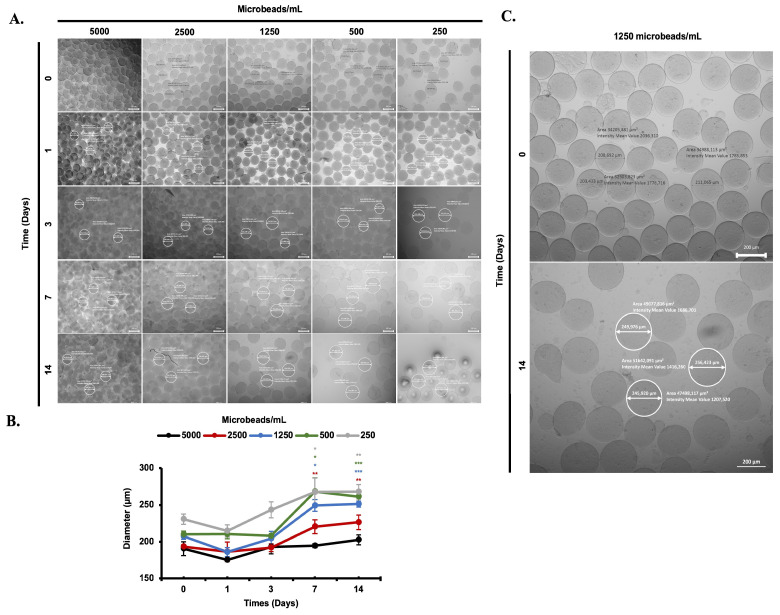
Diameter changes between each group (5000, 2500, 1250, 500, and 250 microbeads/mL) of the experiment are visualized during 14 days of incubation under the light microscope (5×, scale bar: 200 µm) (**A**). Diameter changes between each group of an experiment are graphed during 14 days of incubation under the light microscope (**B**). Diameter changes between day 0 and day 12 of 1250 microbeads/mL are shown (**C**). *: *p* < 0.05, **: *p* < 0.01, ***: *p* < 0.001.

Alginate microbeads were produced with an encapsulator device (Nisco Encapsulator VAR V1 LIN-0043, Nisco Engineering AG, Zurich, Switzerland) and cultured in Mesenchymal Nutrient Free Medium (Biological Industries) during 14 days in medium (Figure 1). A total of 600 microbeads were produced in 30 min, and each group (5000, 2500, 1250, 500, and 250 microbeads/well) were incubated in a 1 mL cell culture medium. The diameter of the microbeads for 12 days of incubation was measured using a fluorescence microscope. The diameter of the microbeads was seen to be increased from 190 to 280 µm day by day for each group of experiments. The diameter of the microbeads was negatively correlated with the number of microbeads per mL, as given in Figure 1.

**Figure 2:**
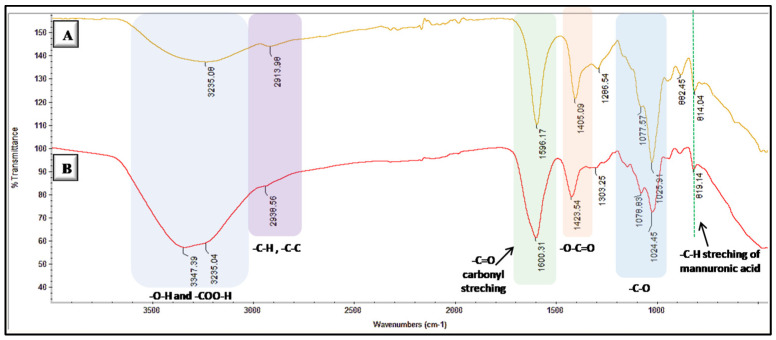
FT-IR spectra for (**A**) pure alginate polymer and (**B**) alginate-based microbeads.

**Figure 3:**
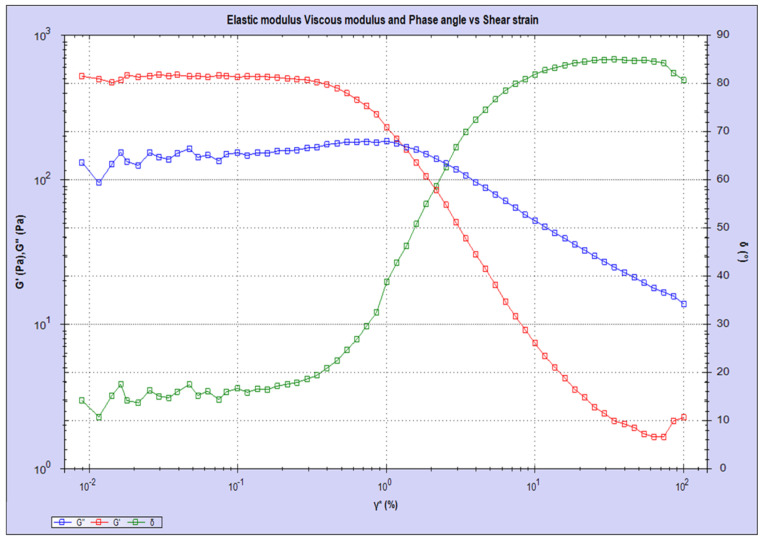
Rheometry results for alginate–based microbeads in swollen state.

The chemical structure of obtained microbeads was confirmed by FT-IR Spectroscopy. As expected, microbeads present all peaks corresponding to characteristic functional groups of alginate polymer (Figure 2). It is clearly seen that the carbonyl peak belonging to the carboxylic acid group of alginates appears at 1600 cm^−1^ and bonds at the backbone of the polymer chains are represented by peaks at 1078 and 1024 cm^−1^ for –C-O and 1423 cm^−1^ for –O-C=O moieties. Furthermore, the degradation behavior of obtained alginate microbeads was investigated by rheometry, where G″ represents viscosity (loss modulus) and G′ represents elasticity (storage modulus). As seen in Figure 3, initial states show the elastic property until the cross point where G″ and G’ overlap, and as G″ and G′ diverge, the deformation in microbead structure happens, and the degradation starts. After that frequency, the stiffness of microbeads starts to decrease, and the G″ value exceeds, pointing to the liquid–like property of the microbeads. This profile indicates the stress-mediated deformation behavior of obtained microbeads.

**Figure 4:**
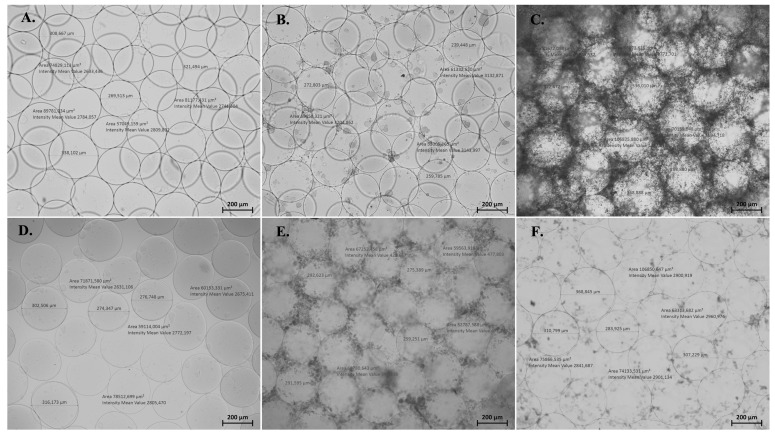
Morphological structure and size of microbeads under light microscope (5×, scale bar: 200 µm) at 37 °C in various incubation conditions (Acetate solution after 1 day (**A**), Medium condition after 1 day (**B**), Phosphate buffer saline (PBS) condition after 1 day (**C**), Acetate solution after 5 days (**D**), Medium incubation after 5 days (**E**), and PBS incubation after 5 days (**F**).

Stability analysis of microbeads and their morphologic evaluations were performed in various incubation conditions (100 mM pH: 5.5 Acetate Solution, Medium, Phosphate buffer saline (PBS), and saline @ 37 °C). The diameter and morphology of the microbeads were compared between each group (Figure 4). No significant change was observed for the diameters of microbeads in different incubation conditions. In some environmental conditions, salt formation around the microbeads was observed) in PBS on day1 (Figure 4C) and in medium on day 5 (Figure 4E). The salt formation was removed via medium and saline wash of the microbeads (Figure 4E,F).

### 3.2. Morphological Evaluation of Cell-Loaded Microbeads

**Figure 5:**
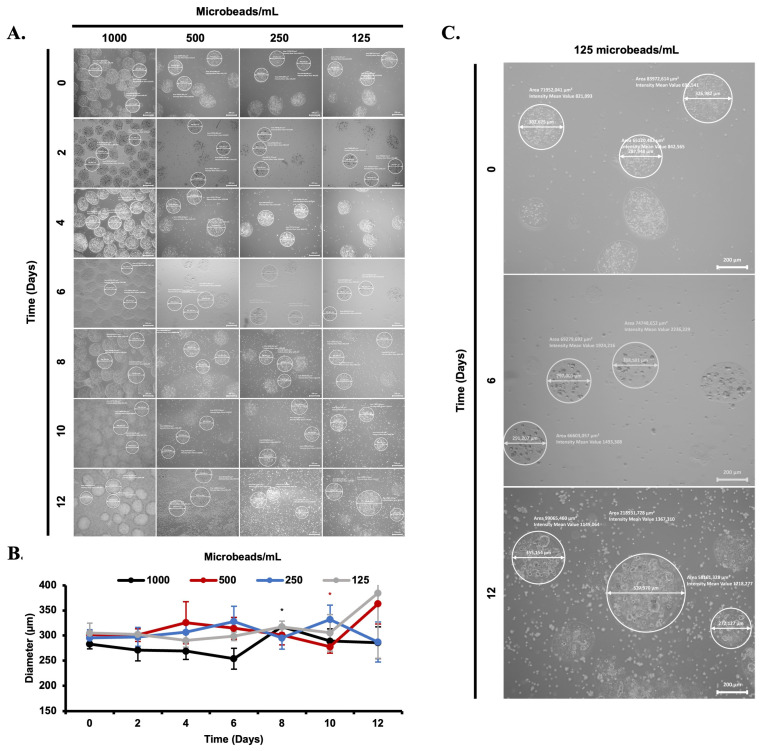
Visualization of L929 release from microbeads time-dependent manner under the fluorescence microscope (5×, scale bar: 200 µm) (**A**). Diameter of L929 loaded beads in MSCs nutrient medium for 12 days period (**B**). Diameter changes between days 0, 6, and 12 for 125 microbeads/mL are shown (**C**). *: *p* < 0.05.

**Figure 6:**
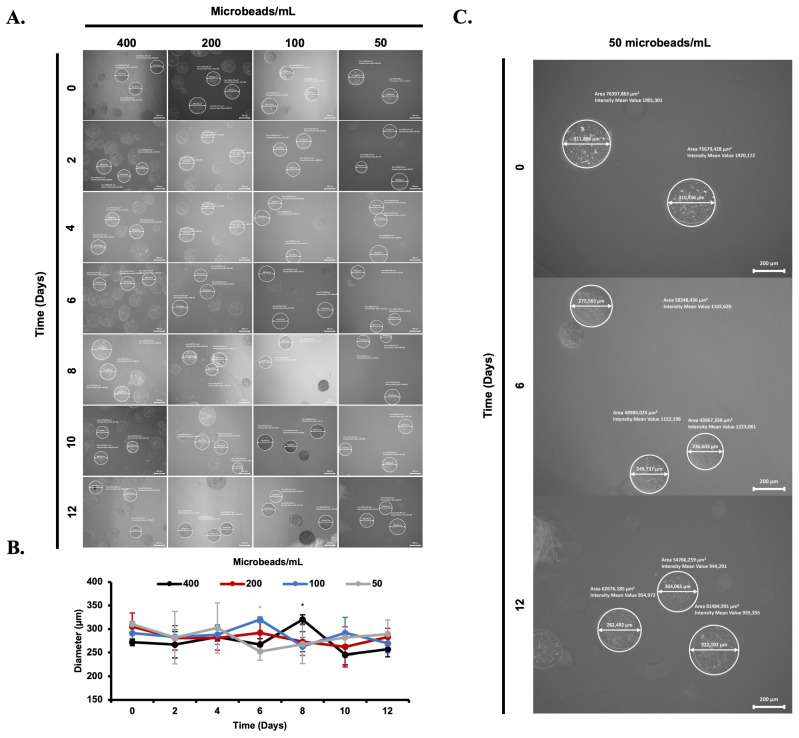
Visualization of Mesenchymal Stem Cell (MSCs) release from microbeads in a time-dependent manner under fluorescence microscope (5×, scale bar: 200 µm) (**A**). Diameter of MSCs loaded beads in MSCs nutrient medium for 12 days period (**B**). Diameter changes between days 0, 6, and 12 of 50 microbeads/mL are shown (**C**). *: *p* < 0.05.

Cell (L929 and MSCs) stability and proliferation profiles were tested with various amounts of microbeads (1000, 500, 250, 125 and 400, 200, 100, 50 microbeads/mL) (Figure 5A and Figure 6A). The diameter of cell-loaded microbeads was measured using a fluorescence microscope and compared with each other. It can be clearly seen that cell release from the alginate microbeads was monitored during the incubation period. The diameter of the microbeads was similar at day 0, whereas on the following incubation days, their diameters were altered in each group of experiments. Non-uniform distribution of the microbeads and cell release were observed under the microscope.

**Figure 7:**
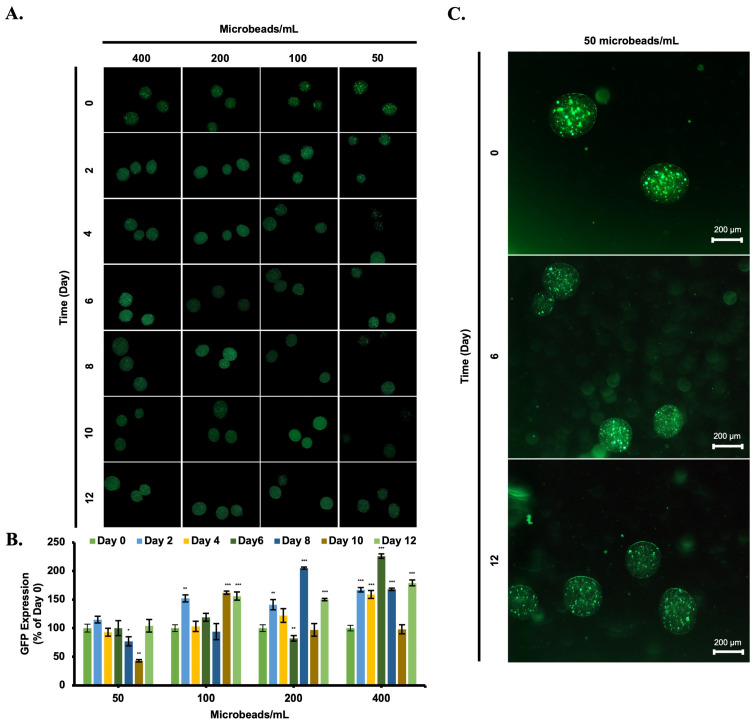
Visualization of green fluorescence protein secretion by Mesenchymal Stem Cell (MSCs) inside microbeads in a time-dependent manner under fluorescence microscope (5×, scale bar: 200 µm) (**A**). Green fluorescence protein intensity by inside mesenchymal stem cells (MSCs) loaded microbeads in a time-dependent manner under fluorescence microscope (5×, scale bar: 200 µm) (**B**). Green fluorescence intensity changes between days 0, 6, and 12 of 50 microbeads/mL are shown (**C**). *: *p* < 0.05, **: *p* < 0.01, ***: *p* < 0.001.

The green fluorescent protein (GFP) signal of mesenchymal stem cells (MSCs) was utilized as a sign not only to monitor cells under a fluorescence microscope but also to understand their metabolic activity during 12 days of incubation (Figure 7A). Changes in the green fluorescence particle (GFP) intensity were observed in each group of microbeads during the incubation (Figure 7B). It was concluded that there was no correlation between the microbeads number and GFP signal intensity. Additionally, fluctuation of the GFP signal was observed for each group of the experiment.

### 3.3. Glucose and Lactic Acid Measurements

**Figure 8:**
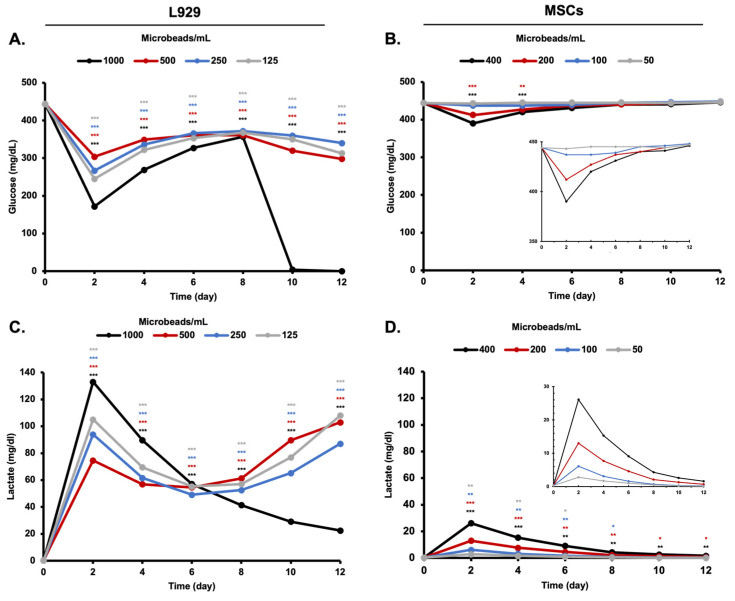
Glucose consumption and lactic acid production of cell line for 12 days. (**A**) Glucose consumption of L929, (**B**) lactic acid production of L929, (**C**) glucose consumption of MSCs, and (**D**) lactic acid production of MSCs. *: *p* < 0.05, **: *p* < 0.01, ***: *p* < 0.001.

Metabolites in the cell culture medium are essential elements for tracking cellular growth. Both cell lines (L929 and MSCs) seem to consume glucose and produce lactate inside their 3D environment. While the glucose consumption level for both cell lines was sharply decreased, the lactic acid production rate was consistently increased in 2 days of incubation. A consistent plot between glucose consumption and lactic acid production in L929 cell media and MSC media was observed for the following incubation time points (Figure 8).

### 3.4. Viable Cells in a Cell Culture Medium Were Observed on Day 12

**Figure 9:**
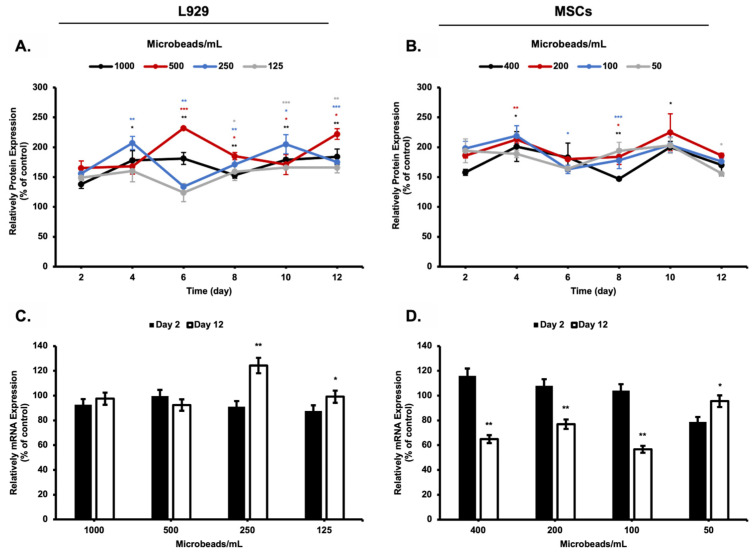
Protein concentrations of cultured medium solution both (**A**) L929 cell line and (**B**) MSCs. mRNA concentrations in medium solutions of (**C**) L929 cell line and (**D**) MSCs. *: *p* < 0.05, **: *p* < 0.01, ***: *p* < 0.001.

The total number of released cells inside the medium and attached to the tissue culture dishes were calculated using a Bio-Rad cell counter device (TC20TM Automated Cell Counter (Hercules, CA, USA)). The cell number of both cell lines (L929 and MSCs cell lines) inside the medium was decreased as the microbead numbers in cell culture media were diminished. The concentration of the cells in the alginate solution was 4 million cells/mL and 2 million cells/mL, respectively. According to the estimated calculations, cell numbers for both cell-loaded microbeads were found to be 48 cells per microbead for L929 cells and 24 cells per microbead for MSCs. These values also seem to be consistent for each group of the experiment (Figure 9). On the other hand, the cell viability (L929 cell lines) gradually decreased with decreasing in the microbeads numbers in cell culture media (67.5, 54, and 60%, respectively, for 500, 250, and 125 microbeads/mL). However, the percentage of the cell viability (MSCs cell lines) was observed to be stable with decreasing in the microbeads numbers in cell culture media (42.5, 45.5, 46.5, and 66%, respectively, for 400, 200, 100, and 50 microbeads/mL).

### 3.5. Total Protein and mRNA Concentration in Culture Medium

**Figure 10:**
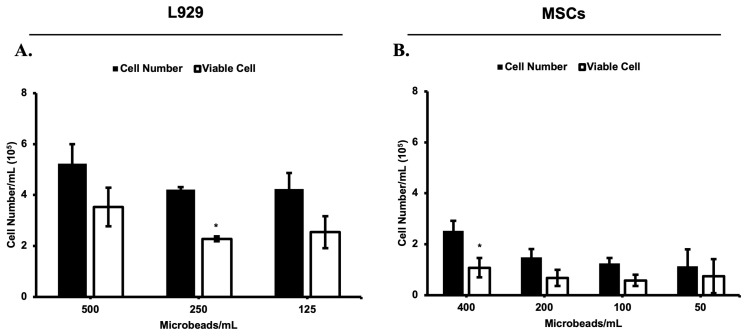
Cell viability ofreleased L929 (**A**) and MSCs (**B**) cell lines in cell culture medium at day 12. *: *p* < 0.05.

Total protein concentration inside the medium sample was measured during 12 days of the incubation period, and changes in the total protein amounts in a cultured medium of L929 and MSCs cell lines were compared with each group of the experiment. BCA assay was used for the quantification of the total protein amount here. The total protein amount inside the medium was found to gradually increase based on the increase in the number of cell-loaded microbeads in the media (Figure 10).

For each group of microbeads, it was analyzed that the mRNA level inside the culture medium of L929 and MSCs was not significantly changed during the incubation period. Only, a relatively small drop was observed in the concentration of the mRNA level inside the culture medium of mesenchymal stem cells (MSCs).

**Figure 11:**
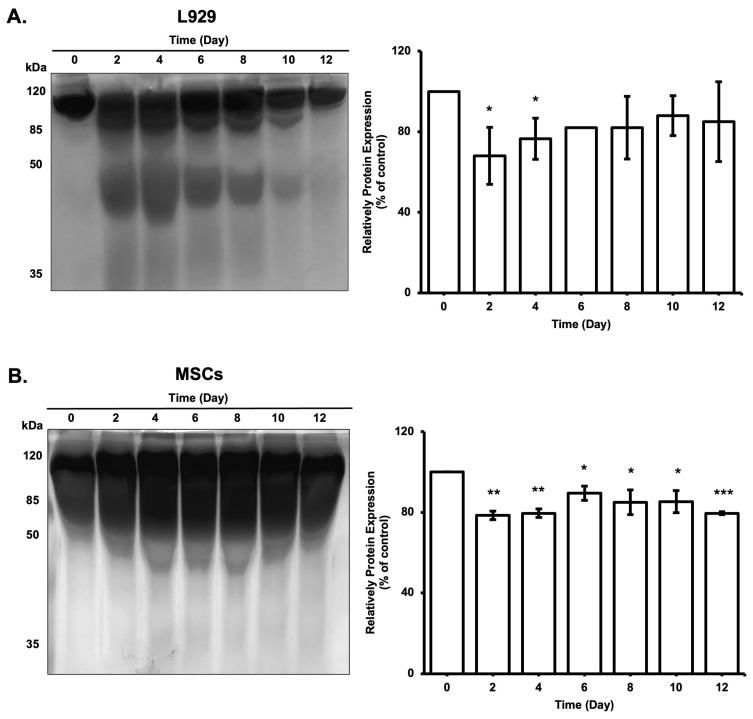
Protein distribution of the cell culture medium of L929 (1000 microbeads/mL) (**A**) and MSCs (400 microbeads/mL) (**B**) during 12 days of incubation. *: *p* < 0.05, **: *p* < 0.01, ***: *p* < 0.001.

Protein distribution of the culture medium was also investigated during the incubation of the cell-loaded alginate via SDS-Page. The protein content profile showed that cells not only continue their metabolic activity during the incubation period but also secrete metabolic proteins into the cell culture medium. Secreted proteins by cells from inside of the microbeads to culture medium seem to be aggregated during the incubation periods, and the aggregation profile of each sample indicates the expression of low abundant proteins on SDS-PAGE (Figure 11). Additionally, consistent results between glucose consumption and protein content inside the culture medium were observed in L929 cells. No difference was observed for MSC cells because of the slower proliferation rate.

## 4. Discussion

At present, MSCs are used widely for the regeneration of various tissue types [20,21]. Their stability and controlled release at the target area stands out as very significant issues for the effectiveness of applied therapy and the resultant regeneration. Previous studies showed that alginate microbeads stay stable in vitro conditions for12 days and also in in vivo conditions for 2 weeks [13]. Leslie and coworkers fabricated injectable alginate microbeads and demonstrated their control enzymatic degradation under various concentrations of alginate lysate. However, they did not consider the stability of alginate lysate at the injection side of the damaged tissue. A mixture of stem cell-loaded microbeads and alginate lysate might not stay together after injection at the target area. So, in this research, we evaluated the non-enzymatical degradation of alginate by the diffusion of calcium out to the environment and the production of the metabolites by cells which were loaded into alginate microbeads.

As an anionic polymer, alginate has a net negative charged group, so the morphology of the alginate microbeads was seen to be affected clearly by environmental pH change. This process mainly occurs through the protonation and deprotonation processes based on the hydrophilicity of the carboxylic acid groups in the anionic polymer. That is why alginate in microbead structure is prone to degradation not only by enzymatic cleavage but also by ion exchange [22]. Salt accumulation was observed in presence of the calcium content after 1-day of incubation in PBS. Similarly, salt-based aggregates were formed in the medium on day 5 of incubation. It is obvious that calcium ions in the alginate solution diffused into the medium and PBS and form a calcium salt inside. The salt content in acetate conditions was seen to be negligible because of the H-bonding in the alginate polymer [23].In cell culture media conditions, alginate microbeads provided a continuous swelling profile due to the presence of chelators, monovalent ions, and non-crosslinking divalent cations such as Mg^2+^ [24,25]. Moreover, the concentration of the CaCl_2_ (75 mM) in the hardening solution is much higher than that in the medium condition (1.8 mM). For this reason, the high-water content and diluted conditions outside the microbeads might probably cause their swelling. Additionally, the size of the microbeads should be lower than 500 µm for injectable applications, and researchers in a previous study fabricated microbeads with a size of 400 µm [13]. However, in this study, alginate microbeads were successfully prepared with a diameter of around 200 µm.

For cell viability in 3D cell culture, the main problem faced is the poor oxygen penetration inside and the absence of metabolites for cellular growth, which is caused by the absence of the vascular structure in this 3D environment. Micro-scale 3D architecture might solve this problem because of the low oxygen gradient from inside to outside. At this point, we have seen that cells inside the microbeads managed to continue their viability during 12 days of incubation. L929 cells in each group of the experiment were found to proliferate inside the microbeads for 7 days and then released into the environment because of the swelling of alginate microbeads. Although the non-uniform distribution of microbeads increased day by day because of the swelling, cell proliferation leads to the formation of a solid spheroid structure inside the microbeads. Moreover, the concentration of cells in alginate solution affects cell–cell interactions, cell proliferation, and cultivation time inside the microbeads during the incubation period [26]. Although MSCs stayed stable inside the microbeads for 12 days of incubation, cell release and debris were observed for 400 microbeads/mL samples on day 12. Their viability profile and proliferation rate were monitored by the expression of green fluorescence-tagged proteins (GFP). GFP signal intensity was detected to be preserved during the incubation period of the cells [27]. Fluctuations of the GFP signal intensity are related to cell doubling and subsequent cell release from the microbeads during the culturing process. Obviously, proliferated cells increased the diameter of the microbeads and especially for fibroblasts, spheroids formation was observed under the light microscope on day 12. Spheroid formation actually indicates that fibroblasts also proliferated inside the alginate microbeads. Cell number and cell viability between each group of the experiment were consistent.

As another indication of the metabolic activity of the encapsulated cells inside the alginate-based microbeads, metabolites were quantitatively analyzed with respect to incubation time. Glucose is an essential element for the metabolic activity of the cell and its concentration affects metabolic processes inside the cell [28]. On the other hand, lactic acid is the metabolic waste product that is produced after the cellular respiration process of the cell. Glucose consumption and lactic acid production levels indicate the metabolism of the cells in in vitro conditions [29]. It is very well known that normoxia, hypoxia, and hyperoxia conditions determined by the soluble oxygen content in water alter the metabolic activity of the stem cells. Glucose consumption of the cells significantly increases during the adaptation of the cells to the environment, especially in hypoxia conditions such as 3D cell culture [30]. Similarly, in this study, glucose consumption of the stem-cell-loaded microbeads significantly increased on day 2. After the adaptation of the cells to the environment, glucose consumption and lactic acid production was gradually linearized like 2D cell culture [26]. After 8 days of incubation, cell number, glucose consumption, and lactic acid production were measured as gradually increasing. Furthermore, alginate degradation seems to be enhanced by large amounts of chelators, monovalent ions, and non-crosslinking divalent cations such asMg^+2^ in a culture medium, restricting the concentration and cultivation time of microbeads in vitro conditions. Lastly, it was obvious that the proliferation of cells increases the pH of the environment through metabolic waste and oxygen demand by the cells.

High acidic conditions cause cell death during the incubation of the cell-loaded microbeads, and this trend might be monitored by glucose consumption and lactic acid production level. During the incubation of the group “1000 microbeads/mL”, glucose consumption was found to be significantly dropped, and lactic acid production was accordingly decreased in relation to environmental conditions such as pH or a large amount of metabolic waste. This result has not been seen in other experimental groups (500, 250, and 125 microbeads/mL), though.

Various hormones, growth factors, and proteins were used to regulate the differentiation and proliferation of cells in culture media. Metabolic activities of the cells are not only dependent on cultivation conditions but also affected by their protein expression [26,31]. Cells induce protein expression to adapt to the environment and continue metabolic activity during cultivation. Protein profiles investigated in this research show that protein concentration inside the culture medium increases day by day, but glucose consumption and lactic acid production provide an approximately linear curve. These results indicate that protein expression inside the microbeads was not regulated by glucose consumption and lactic acid production of the cells.

MSC-loaded alginate microbeads have shown great application potential not only in regenerative therapy but also in various therapeutic approaches. Biodegradability of the alginate microbeads seems to contribute not only to the releasing of the cells out of the microbeads to the environment but also to the liberation of therapeutic proteins to the degenerative side. According to the obtained results, released cells stayed viable at 12 days of incubation in all groups of the experiment, yet cellular activity inside the microbeads construct was preserved, as well.

## 5. Conclusions

This study comprises the construction of injectable cell-loaded microbeads as stable and efficient delivery platforms for in vitro cell culture experiments. This platform is potentially applicable to cell therapeutic approaches based on the results of its stability, size(190–280 µm range), and biodegradability. We performed a comprehensive optimization study for both the fibroblasts (L929 cell line) and MSCs and prepared their alginate microbead-loaded versions under in vitro conditions. The metabolic activity of these cells was tracked by various experimental methods (*glucose-lactic acid measurement*, *GFP intensity calculation*, *SDS page*). During 12 days of incubation, L929 and MSCs constitute spheroid formation inside the microbeads, while both cell lines seem to preserve their viability, cellular functioning, and metabolic activity at a higher level. In this way, cells were not only stabilized in the microbead structure for the initial incubation time but also released into the environment afterwards by the degradation of the polymeric wall of microbeads. In addition, the protein expression levels were observed consistently increased from 68% to 85%, with the proliferation of fibroblasts encapsulated inside from day 2 to 12. These findings clearly indicate that microencapsulation of living cells under optimized conditions has a strong impact on providing a powerful tool for ensuring the survival and metabolic activity of cells to be delivered to the injured or damaged tissue part for cellular therapy purposes.

## Data Availability

The data used to support the findings of this study will be available from the corresponding author upon request.
